# Australian Women’s Responses to Breast Density Information: A Content Analysis

**DOI:** 10.3390/ijerph20021596

**Published:** 2023-01-16

**Authors:** Tanvi Pandya, Zixuan Liu, Hankiz Dolan, Jolyn Hersch, Meagan Brennan, Nehmat Houssami, Brooke Nickel

**Affiliations:** 1 Sydney School of Public Health, Faculty of Medicine and Health, The University of Sydney, Sydney, NSW 2006, Australia; 2 Westmead Clinical School, Faculty of Medicine and Health, The University of Sydney, Sydney, NSW 2145, Australia; 3 The National School of Medicine, The University of Notre Dame Australia, Sydney, NSW 2007, Australia; 4 The Daffodil Centre, The University of Sydney, a Joint Venture with Cancer Council NSW, Sydney, NSW 2006, Australia

**Keywords:** breast density, mammography, communication, women’s health

## Abstract

Breast density (BD) is an independent risk factor for breast cancer and reduces mammographic sensitivity. This study explored women’s responses and intentions if notified that they had dense breasts. Methods: Content analysis was used to assess responses from a written questionnaire undertaken in conjunction with focus groups on BD involving 78 Australian women aged 40–74. Results: Half the women reported that they would feel a little anxious if notified they had dense breasts, while 29.5% would not feel anxious. The most common theme (29.5%) related to anxiety was the psychosocial impact of the possibility of developing cancer, and women believed that being better informed could help with anxiety (26.9%). When asked what they would do if notified of having dense breasts, the most common response was to consult their doctor for information/advice (38.5%), followed by considering supplemental screening (23%). Consequently, when asked directly, 65.4% were interested in undergoing supplemental screening, while others (10.3%) said they “wouldn’t worry about it too much”. Discussion: These findings have important implications for health systems with population-based breast screening programs that are currently considering widespread BD notification in terms of the impact on women, health services and primary care.

## 1. Introduction

Breast density (BD) refers to the proportion of fibrous and glandular breast tissue compared to fatty tissue in the breast, as measured on a mammogram [1,2]. A mammography report classifies breast density into four categories reflecting whether the breasts (A) are entirely composed of fatty tissues, (B) have areas of dense tissue scattered throughout, (C) are evenly dense, or (D) are extremely dense [2]. Women whose breasts are reported to be in the last two categories (C and D) are said to have dense breasts [3]. Dense breasts are common, estimated to be present in 40–50% of women who are part of the breast-screening population in the United States (USA) [3], and at least 23% of Australian women of breast-screening age (i.e., aged 40+) [4]. Women with dense breasts have an increased risk of breast cancer as BD is one of a number of independent risk factors [5]. Furthermore, having dense breasts increases the chances of cancer being missed during screening as it reduces the sensitivity of a mammography by masking the tumour and making it difficult to detect [5]. This increases the risk of a false negative result or an interval breast cancer during cancer screening [6].

Information about BD is not routinely provided to women through breast screening programs except in the USA, meaning women in many countries are unaware about their BD. In 2019, the USA passed national BD legislation requiring screening services to include BD information in every mammogram report, as a way of providing women with more information about their health while trying to manage the risk of breast cancer [7]. This national mandate to notify BD has not happened in other countries; however, there are some international jurisdictions now notifying women, and a recent European consensus paper from an imaging group recommends breast density notification after a mammogram as well as biennial MRC screening for women with extremely dense breasts [8]. In 2020, the BreastScreen Australia Standing Committee on Screening stated that “Until more evidence is available on how breast density is best assessed and managed, … BreastScreen Australia should not routinely record breast density or provide supplemental testing for women with dense breasts” [9].

Supplemental screening, such as ultrasound and magnetic resonance imaging (MRI), in women with high BD may help detect early-stage breast cancers [10] and BD notifications may encourage women who are at increased risk to seek supplemental screening [11]. When used in combination with a mammography, these supplemental imaging modalities may increase breast cancer detection; however, these could also give false positive results and increase the risk of overdiagnosis [12].

Currently, there is little evidence regarding responses and intentions of women surrounding the topic of BD outside of the USA [13]. A crucial knowledge gap remains in terms of understanding how women view the topic of BD. Understanding the beliefs of women may have an impact in countries such as Australia, where there is a strong push from advocacy groups to implement a national BD notification system [14]. This research aims to explore the anxiety that Australian women may experience if they were notified that they had dense breasts, what actions they may take, and their interest in supplemental screening.

## 2. Materials and Methods

### 2.1. Study Design

This study used content analysis [15]. This method combined elements of both qualitative and quantitative methods to assess and analyse written free-text data from Australian women who participated in an online focus group [16]. The community-based online focus group study, which was approved by the University of Sydney’s Human Research Ethics Committee (2020/160), administered written questionnaires (using Qualtrics) to the participants at the start and end of the session. Seventy-eight Australian women between the ages of 40–74 without a personal diagnosis of breast cancer or ductal carcinoma in situ (DCIS) participated in the focus groups. They were recruited by an independent research recruitment organisation (Taverner research). The focus groups were conducted online, utilizing Zoom videoconferencing software in September 2020. Focus groups were stratified according to age (40–49, 50–59, 60–69, and 70–74) and consisted of approximately six participants per group. Participants were from either New South Wales or Queensland, and two focus groups per age group were determined in each state (except for the 70–75 age group, which had one group per state).

Each session comprised an introduction to the topic, a demographic questionnaire, warm-up discussions, detailed presentations interspersed with group discussions, and a final questionnaire [16]. The information provided during the sessions included pictures and infographics describing BD and its implications. The presentations also talked about the risk of overdiagnosis and overtreatment correlated to supplemental screening, and presented women with two hypothetical case studies of women with dense breasts making supplemental screening decisions. These sessions incorporated an opportunity for women to share their thoughts, feelings and questions in response to the information presented. In this study, women’s responses to Likert, multiple choice and free-text questions on BD notification in terms of (a) anxiousness, (b) actions they anticipated, and (c) their intentions for seeking supplemental screening from final questionnaire data were analysed descriptively and using a content analysis method. Please see Supplementary List S1 for a complete list of the questions that were analysed.

### 2.2. Analysis

The recorded focus group discussions were analysed to identify recurring themes and patterns using thematic analysis. Findings from the focus group discussions are reported elsewhere [16]. Participants’ questionnaire responses were compiled into a password-protected Excel file. Researchers (TP, ZL, and BN) independently assessed all responses and continuously looked for similarities and recurring patterns in the data. The data were then categorized into an initial coding framework for each independent variable (anxiety, responses to breast density information and interest in supplemental screening) to identify repeating concepts and major themes. The researchers then proceeded to code the responses into their suitable category, and responses could be coded into more than one primary theme and/or sub-theme. Not every response was coded into a sub-theme. The coding framework was then checked for validity and reliability of the data. Cohen’s Kappa statistic was used to check for interrater reliability for the coded data [17], and was run with BN. The result was 0.83 for anxiousness regarding BD notification, and 0.97 for responses to breast density information and interest in supplemental screening, indicating almost perfect agreement between the researchers.

## 3. Results

Seventy-eight women took part in the study (Table 1). Of these, 70.5% were of screening target age (50–74 years), 30.8% were born overseas, and 18% had their highest educational qualification at the high school level or below, indicating that this sample had a higher level of education compared to the general Australian population [18]. Thirty-one (39.8%) women lived in regional or remote areas, which is higher than the 27.1% of the general Australian population living in regional or remote areas [19].

The sample reported high general wellbeing and low levels of cancer worry. Around 85% of women had experience in breast screening. Among them, screening through the publicly funded screening program BreastScreen was most common (67.9%). Few participants among women in their 40s reported proactively initiating screening or receiving a mammography, as they had not reached the target age of 50–74 to receive invitations for screening.

### 3.1. Anxiety

When asked how anxious women believed they would feel if they were notified of having dense breasts, 39 women (50%) indicated that they would feel a little anxious if told that they had dense breasts, 10 women (12.8%) would feel moderately anxious, and 4 women (5.1%) would feel very anxious. However, 23 women (29.5%) indicated that they would not feel anxious at all.

Table 2 summarises the nine primary themes and possible sub-themes identified in the women’s responses regarding anxiety and dense breasts.

Some participants’ quotes have been selected to demonstrate the respective theme and these are presented in the tables below. The frequency represents the number of women who provided a particular response.

The most common theme (29.5%) was women expressing views about the psychological impacts of being informed about their breast density, with 19.2% of these women mentioning they worried specifically that dense breasts may increase their chances of developing breast cancer. A similar proportion (26.9%) of women mentioned that they would be better informed regarding their health, regardless of their anxiety. On the other hand, 15.4% of women mentioned that they would not worry about breast density. A small proportion (12.8%) of women held the view that dense breasts are an important risk factor when it comes to breast cancer and that is what would make them anxious.

### 3.2. Responses to Breast Density Notification

The thematic analyses identified seven major themes in the data related to the women’s intentions following hypothetical BD notification, shown in Table 3.

Many women expressed that receiving information about their breast density would make them more vigilant towards their breast health and would encourage them to seek additional screening (23%). The most common reaction from women when asked what they would do if they were notified of having dense breasts was to communicate with their doctor/GP and follow their advice. This view was shared by 38.5% of the participants and emerged as a major theme in this context. The desire to be guided by their doctors and healthcare professionals came from wanting to seek and gather further information about false positives and overdiagnosis. Some women opted towards increasing the frequency of having mammograms (15.4%). Other than that, 20.5% of women indicated an intention to educate themselves regarding the topic by doing more research and joining support groups to gain as much information as possible. The other 10.3% conveyed that they would become more ‘judicious’ and ‘vigilant’ with self-examination. A total of 19.2% of women suggested that they would not change anything and would do ‘nothing more’ than what they already do now.

### 3.3. Interest in Supplemental Screening

When asked about their interest in supplemental screening, the majority of women (65.4%) were interested, while 24.4% of women reported that they were unsure if they would be interested in supplemental screening if they had dense breasts, and the remainder of women (10.3%) reported no interest.

Table 4 outlines the thematic analysis for interest in supplemental screening. The thematic analysis identified seven major themes, with sub-themes emerging from some of them.

The majority of women (62.9%) responded in favour of additional supplemental screening; the primary reason was wanting to gain knowledge about their bodies. The willingness to undergo further testing was greatly emphasized by ‘peace of mind’, ‘taking precautions’ and ‘reassurance’ regarding their health. A small percentage of women (9%) voiced concerns about the risks of overdiagnosis and overtreatment related to supplemental screening. They expressed their desire for more information before making any further decisions. For others (5.1%), the extra costs for additional screening were a barrier towards supplemental screening. For 7.7% of the participants, this issue was less concerning and they expressed a more relaxed attitude towards the topic of supplemental screening.

## 4. Discussion

This analysis of focus group participants’ responses and intentions after being presented with detailed BD information suggested that the majority of Australian women would feel some level of anxiousness if they were notified of having dense breasts. This was mainly driven by the psychological impact of the possibility or increased risk of cancer. In previous studies, it has been shown that providing breast density notification and accompanying information can improve knowledge about the reduced sensitivity of mammography in dense breasts and the increased risk of breast cancer; however, this may also increase women’s anxiety and confusion [13,21]. In order to mitigate any unintended outcomes, such as anxiety, the phrasing of the BD notifications should be carefully crafted to enhance the understanding of the notifications so that the women are accurately informed [22]. In a quantitative study conducted in the USA, anxiety was reported to be the driving factor towards women’s intentions for additional supplemental screening, emphasising the significance of anxiety in potential decision making [23]. In the present study, many women reported greater anxiety if they also reported other risk factors for breast cancer, such as family history. Some women also reported that the reduced sensitivity of the mammogram would make them anxious. On the other hand, there were women who believed that BD notification would help them stay more aware regarding their bodies and be more informed about their breast health.

When the women were asked what they would do upon being notified of having dense breasts, most women said that being guided by their doctors and obtaining their recommendations would be the first step they would take, followed by an inclination towards additional screening. An Australian study from the state of Western Australia where they currently notify women of their breast density found that half of women notified consulted or intended to consult with their general practitioner (GP), and of those, 50% were referred for supplemental screening and 20% reported having an ultrasound [24]. Of concern, however, is that important gaps still exist in GP knowledge about breast density and confidence in having discussions with women about the implications [25,26], and the current lack of evidence-based management guidelines for GPs.

Interestingly, in our study, a few women would favour having mammograms on a yearly basis rather than having them every two years. This could be due to the downsides of supplemental screening presented to the participants and may reflect a lack of understanding of the potential harms of mammography screening for women with high BD. Annual mammograms in women with dense breasts at average risk are not likely to improve outcomes compared to biennial screenings, in terms of the balance between harms and benefits of screening [27]. Furthermore, additional findings from these focus groups reported elsewhere [16] also demonstrated that women emphasised the ‘right to know’ about any information regarding their bodies and felt that BD should be measured and reported as part of the routine screening.

In general, women opted for additional supplemental screening for their own reassurance. These findings were consistent with some of the common themes that emerged in this research: wishing to be informed about their body, taking precautions, finding peace of mind, and being able to rule out cancer. It also indicated that despite being presented with potential drawbacks of supplemental screening including the increased risk of false-positives and overdiagnosis, most women prefer to focus on true-positives, and would rather be overdiagnosed than underdiagnosed when it comes to breast cancer. This was not surprising as most adults believe that routine cancer screening is always a ‘good idea’ as finding the cancer early saves lives [28]. For other women, however, the decision was heavily dependent on the existing screening results or on whether they had any symptoms. It should also be noted that harms related to false-positives and overdiagnosis were the primary reasons for women not wanting to proceed with supplemental screening should they be notified of having dense breasts. False-positive results are much more common in women who have dense breasts, and about half of the women getting screened over a 10-year period could have had a false-positive report at some point [29,30]. The unfavorable reactions to a false-positive result, coupled with physical discomfort and potential out-of-pocket costs that was discussed amongst the women during the focus group, likely influenced this response.

This research has both limitations and strengths. This study was conducted only in Australia. Hence, forming generalized conclusions about all women around the globe is not possible, and research conducted in other regions may not be comparable. However, similar findings in studies from the USA [13] provide reassurance that the findings are more generalizable. The study sample was also demographically reasonably representative of the Australian population. It should be noted that there was a small sample size of 78 women for this research. Moreover, women’s responses were short and were recorded online. This leads to some ambiguity among any conclusion driven from those responses. Therefore, the research team predicts that in-person responses will lead to different and more varied results. Despite the limitations, there were multiple strengths of the project. Active participation by the women involved in this study increased their awareness towards breast density. Furthermore, the researchers indicated almost perfect agreement (Cohen’s Kappa). This demonstrates the results are highly reliable and have internal validity in terms of the coding method.

## 5. Conclusions

It is important that health systems with population-based breast screening programs currently considering the impacts of widespread BD notification are aware that informing women about having dense breasts using evidence-based information may still pose a risk of anxiety for some women, while for others this may be a valued opportunity to gain more knowledge about their bodies. Furthermore, GPs and other clinicians involved in breast care would likely be the first point of contact if women were notified of having dense breasts, and there may be a high interest in undergoing additional supplemental screening. Therefore, these clinicians would need training and support to provide advice about breast density to women, if breast density notification becomes widespread across Australia.

## Figures and Tables

**Table 1:** Participants’ characteristics (*n* = 78) *.

Characteristic or Response	No. of Participants *n* (%)
**Age**	
40–49	23 (29.5)
50–59	23 (29.5)
60–69	21 (27)
70–75	11 (14)
**Marital Status**	
Single	19 (24.4)
Married/living with a partner	41 (52.6)
Divorce/separated/widowed	18 (23)
**Aboriginal and/or Torres Strait Islander origin**	2 (2.6)
**Birth Place**	
Australia	54 (69.2)
New Zealand, UK, or USA	16 (20.5)
Other	8 (10.3)
**Years since moving to Australia (*if born overseas*)** **	
<10	3 (3.8)
10–29	2 (2.6)
>30	17 (21.8)
**State of residence**	
New South Wales	41 (52.6)
Queensland	37 (47.4)
**Rurality**	
Urban	47 (60.2)
Regional	30 (38.5)
Remote	1 (1.3)
**Highest educational qualification**	
University Degree	37 (47.4)
Diploma or Certificate	27 (34.6)
Higher School Certificate or equivalent	8 (10.3)
School Certificate or intermediate or equivalent	6 (7.7)
**Employment Status** **	
Full time	5 (6.4)
Part time	36 (46.2)
Retired	16 (20.5)
Student or other	20 (25.6)
**WHO-5 Well-Being Index Score (Mean (SD))** ^†^	66.6 (17)
**General self-rate health**	
Excellent, very good, good	68 (87.2)
Fair or poor	10 (12.8)
**Family history of breast cancer**	
Yes	15 (19.2)
No	63 (80.8)
**Worry about developing breast cancer**	
Not worried at all	21 (26.9)
Worry a little	50 (64.1)
Quite worried	6 (7.7)
Very worried	1 (5.1)
**Breast screening rounds attended**	
None	12 (15.4)
Once	16 (20.5)
Twice	6 (7.7)
Three times	6 (7.7)
Four or more times	38 (48.7)
**Time since last screening (if screened)**	
Within the last 2–3 years	54 (69.2)
3 years ago, or more	12 (15.4)
**Screening services used (if screened)**	
BreastScreen (publicly funded screening program)	53 (67.9)
Private screening service	13 (16.7)
**Been previously told or notified of BD**	
Yes	9 (11.5)
No	69 (88.5)

* Adapted from Nickel et al., 2022 [16]. Data comes from the demographic questionnaire administered at the start of the focus group session. ** Missing data. ^†^ A score of 0 represents the worst possible wellbeing and 100 represents the best possible wellbeing [20].

**Table 2:** Main themes and sub-themes surrounding the topic of anxiety related to information about dense breasts with quotations and frequencies (*n* = 78) *.

Theme Description	Example	Frequency (%)
1. Psychological impacts	‘Getting cancer scares me’	23 (29.5)
1a. Anxious, worried, or concerned	‘I worry about everything, and I am anxious when it comes to my health as I know I don’t look after myself very well’	8 (10.3)
1b. Increases the risk/possibility of breast cancer	‘It may increase my chances of getting breast cancer’	15 (19.2)
2. Better informed	‘I’m now far better informed as a result of this educational session and discussion’	21 (26.9)
2a. Aware of possibility of breast cancer/improve breast health	‘I would be more aware of the possibility of breast cancer and their options’	7 (9.0)
2b. Reduce risk of breast cancer	‘Would want to know exactly what steps I need to take to ensure my health and safety’	3 (3.8)
2c. More vigilant	‘I have already been told this and it did not cause anxiety. Now that I know what it actually means it will just make me more vigilant.’	6 (7.7)
3. Would not worry/not a big deal	‘I don’t worry about things at the best of times’	12 (15.4)
4. Increased risk due to other risk factors for breast cancer	‘As I’m aware it’s a risk factor for Breast cancer’	10 (12.8)
4a. Family history	‘Due to my background and family history of breast cancer’	4 (5.1)
4b. Increase with age	‘…a higher risk to cancer but knowing it is usually an age issue I would not be highly’	1 (1.3)
5. Mask cancer/harder to detect	‘It could potentially make it harder to detect cancer’	9 (11.5)
6. Regular screening/further testing	‘While I can have more thorough screening it is something that I can do little about’	6 (7.7)
7. False positives	‘I know now that the breast density mammogram test could be also false positive’	3 (3.8)
8. Talk to/discuss with doctors	‘Would need to discuss this more with my Dr to find out more’	2 (2.6)
9. Miscellaneous/cannot be coded		6 (7.7)

* Responses could be coded to more than one theme and sub-theme.

**Table 3:** Main themes and sub-themes for women’s intentions following hypothetical BD notification with quotations and frequencies (*n* = 78) *.

Theme	Example	Frequency (%)
1. Talk to the doctor/follow doctor’s advice	‘I would get the advice of my doctor and information first’	30 (38.5)
2. Additional screening—supplemental (ultrasound, MRI), i.e., beyond mammography)	‘If recommended, have further testing e.g., MRI or ultrasound’	18 (23)
3. Educate oneself—do more research/find out more information	‘I’d research and look for support groups, contact people who’d been through breast cancer and get more information.’	16 (20.5)
4. Nothing different, i.e., continue regular screening	‘Nothing more than I do now’	15 (19.2)
5. More frequent mammograms, i.e., every year	‘Have yearly mammograms’	12 (15.4)
6. Do regular self-checks and examine breasts	‘Be more judicious with self-examination’	8 (10.3)
7. Not sure what I would do	‘Not sure’	2 (2.5)

* Responses could be coded to more than one theme and sub-theme.

**Table 4:** Main themes and sub-themes for supplemental screening with quotations and frequencies (*n* = 78) *.

Theme	Example	Frequency (%)
1.Wish to be informed/knowledge about their body	‘To find out more, to know and to take charge of what is best for my body under current medical advice’	49 (62.9)
1a. Want to know the situation, for reassurance	‘I’d feel more reassured if I had as many tests available as possible’	7 (9)
1b. Want to be informed to rule out cancer	‘To ensure that cancer was not present’	12 (15.4)
1c. Precaution	‘To ensure that I had the best available screening opportunities to avoid getting breast cancer’	11 (14.1)
1d. Peace of mind	‘For my own mental thoughts’	5 (6.4)
2. Dependent on breast screening results	‘I would have an MRI if I had a lump shown in the scan’	9 (11.5)
2a. If they had symptoms	‘Depends if felt I had other symptoms’	5 (6.4)
3. Harms	‘It would depend on my doctor’s recommendation and other risk factors (or lack thereof) to balance up whether supplemental imaging would be more beneficial or not. Need to consider false positives and over diagnosis, which are more likely with supplemental imaging. At this point I don’t believe I am at high risk of breast cancer, so one risk factor (breast density) may not be enough to do supplemental, but if I had further risk factors or my doctor recommended, then I would do the supplemental.’	7 (9)
3a. Risk of overdiagnosis	‘I do not like the idea of over diagnosis’	5 (6.4)
3b. False positives	‘The risk of false positives really concerns me—especially in relation to unnecessary treatment’	3 (3.8)
4. Not an issue/not concerned or worried	‘Because it would be just one factor and at the moment it is not an issue for me’	6 (7.7)
5. Out-of-pocket costs	‘Concern about potential financial costs’	4 (5.1)
6. Age dependent	‘The older I get yes, but younger no’	3 (3.8)
7. Miscellaneous/cannot be code		4 (5.1)

* Responses could be coded to more than one theme.

## Data Availability

Data are available upon reasonable request.

## Supplementary Materials

The following supporting information can be downloaded at: https://www.mdpi.com/article/10.3390/ijerph20021596/s1, List S1: List of questions included in this analysis.
