# Supplementary material for: Australian Women’s Responses to Breast Density Information: A Content Analysis
Source: Int J Environ Res Public Health. 2023 Jan 16;20(2):1596. doi: 10.3390/ijerph20021596 (PMC9861812; doi:10.3390/ijerph20021596)
Supplement: Supplementary file 1 [file ijerph-20-01596-s001.zip › ijerph-2080727-supplementary.pdf]

Supplementary List S1. List of questions included in this analysis

How anxious do you think you would be if you were told you had dense breasts?

☐ Not at all anxious

☐ A little anxious

☐ Moderately anxious

☐ Very anxious

☐ Extremely anxious

☐ Unsure

Why?

____

____

What do you do think you would do if you were notified you had dense breasts?

____

____

____

____

Why?

____

____

Do you think you would be interested in supplemental (additional) imaging (e.g. MRI, ultrasound) if you had dense breasts?

☐ Yes

☐ No

☐ Unsure

Why?

____

____
